# Unveiling MAGEA3: a novel predictive biomarker for bevacizumab resistance in colorectal cancer

**DOI:** 10.20517/cdr.2025.35

**Published:** 2025-04-28

**Authors:** Juncheng Su, Jiahui Wang, Weilin Chen, Yingjie Xu, Wen Yang, Weiwei Liu, Zheng Wang, Masha Huang

**Affiliations:** ^1^Department of Gastrointestinal Surgery, Renji Hospital Affiliated, Shanghai Jiao Tong University School of Medicine, Shanghai 200127, China.; ^2^Department of Biochemistry and Molecular Cell Biology, Shanghai Key Laboratory for Tumor Microenvironment and Inflammation, Shanghai Jiao Tong University School of Medicine, Shanghai 200025, China.

**Keywords:** Colorectal cancer, bevacizumab resistance, MAGEA3

## Abstract

**Aim:** Bevacizumab has long been a cornerstone in the treatment of colorectal cancer (CRC), serving as a fundamental antiangiogenic therapeutic option. However, a significant proportion of patients exhibit insensitivity to bevacizumab, and no reliable biomarker has been established to predict treatment efficacy. Notably, while many angiogenic factors in tumors have been extensively studied, they have failed to consistently demonstrate reliable predictive value for patient survival outcomes in CRC. This study is designed to screen tumor biomarkers with predictive value for bevacizumab resistance in CRC.

**Methods:** Online CRC databases with bevacizumab treatment were downloaded from the GEO datasets along with the TCGA database, which were then analyzed to generate genes overexpressed in bevacizumab non-responders. In vitro experiments using colorectal cancer cell lines were then performed to explore the underlying mechanism of the candidate gene that impacts bevacizumab efficacy. Finally, clinical samples of CRC were collected to validate the predictive effect of the candidate gene on bevacizumab efficacy.

**Results:** We conducted comprehensive analyses of CRC patient datasets, identifying MAGEA3 as a pivotal gene that is not only highly upregulated in bevacizumab-resistant primary CRC but also strongly associated with poor overall survival prognosis. Our in vitro experiments revealed a novel mechanistic insight: MAGEA3 specifically inhibits the expression and secretion of VEGF through the mTOR signaling pathway in colorectal cancer cells, while exhibiting minimal impact on other key angiogenic factors such as PDGF, FGF, and ANGPT2. This selective regulation of VEGF provides a molecular basis for MAGEA3's role in bevacizumab resistance. Furthermore, we discovered that MAGEA3 significantly impairs mitochondrial function in cancer cells, suggesting an additional layer of complexity in its oncogenic role. Clinically, our findings demonstrated that high baseline levels of MAGEA3 in CRC patients were strongly associated with worse progression-free survival (PFS) following bevacizumab treatment.

**Conclusion:** Collectively, these findings position MAGEA3 as a promising predictive biomarker for bevacizumab resistance in CRC, offering a potential solution to the longstanding challenge of treatment stratification.

## INTRODUCTION

Colorectal cancer (CRC) remains a leading cause of gastrointestinal cancer-related morbidity worldwide, with persistent challenges in treatment outcomes^[1]^. Antiangiogenic therapy is one of the earliest targeted therapies for CRC^[2]^. Dating back to the 1970s, when angiogenesis was first recognized as a hallmark of cancer, its clinical benefits have remained variable^[3]^. Bevacizumab, an anti-vascular endothelial growth factor (VEGF) monoclonal antibody approved for CRC in 2004^[2]^, demonstrates primary resistance in a significant proportion of patients^[4]^. However, common pathological features of CRC, such as KRAS and p53 mutation status, have failed to predict the efficacy of bevacizumab, underscoring the urgent need to elucidate the underlying mechanisms and identify reliable predictive markers of bevacizumab resistance^[5-7]^. Resistance to bevacizumab has been associated with multiple underlying mechanisms, including the adaptive transformation of cancer cells induced by hypoxia, the presence of alternative angiogenesis signaling pathways, and dynamic tumor-stromal cell interactions^[8-10]^. Numerous clinical studies have investigated the potential predictive effects of in situ and circulating angiogenic factors (CAFs) on bevacizumab efficacy in CRC^[11]^. However, the results have shown that the predictive value of these factors is weak and inconsistent across different trials^[12]^. Moreover, the expression levels of these factors fluctuate during bevacizumab treatment, rendering them unsuitable as stable biomarkers for predicting efficacy. These limitations highlight the critical need for more robust and stable biomarkers to guide bevacizumab therapy in CRC.

Melanoma-associated antigen 3 (MAGEA3) is a member of the class I melanoma antigen (MAGE) family proteins belonging to the cancer-testis antigens^[13]^. It is restrictively expressed in germline and trophoblast lineage cells under normal conditions but exhibits abnormally high expression in some tumors, including melanoma, brain tumor, breast cancer, lung cancer and CRC, partially due to hypomethylation of its promoter region^[13-16]^. MAGEA3 has varying effects on the phenotype of different tumor cells. Studies have reported that MAGEA3 can promote proliferation or inhibit apoptosis of various cell lines of melanoma, CRC, gastric cancer, and cervical cancer^[14,16-18]^. Additionally, MAGEA3 can inhibit apoptosis of pancreatic cancer cell lines under conditions of nutritional deficiency^[19]^. However, MAGEA3 appears to inhibit cell proliferation in the glial cell line U57^[20]^. These results indicate the diversity of its functions. Regarding angiogenesis, it has been reported that the simultaneous overexpression of CALR (Calreticulin) and MAGEA3 by adenovirus in the human umbilical vein cell line HUVEC can inhibit angiogenesis, but the role of MAGEA3 has not been described independently^[20]^.

In this study, we identified oncogenes associated with prognosis and bevacizumab resistance in CRC patients through data screening and found MAGEA3 as a potential candidate. We then explored the possible mechanisms by which MAGEA3 contributes to CRC progression and therapy resistance. Finally, clinical sample validation confirmed that high MAGEA3 expression is correlated with poorer prognosis in CRC patients. Given the urgent clinical need for reliable biomarkers to predict treatment response and improve bevacizumab efficacy, our findings suggest that MAGEA3 may serve as a valuable prognostic marker and a potential therapeutic target in CRC.

## METHODS

### Data collection and differential gene analysis

The gene expression dataset GSE60331 was downloaded from the GEO datasets (https://www.ncbi.nlm.nih.gov/geo/) along with the corresponding clinical information. Patients were classified as bevacizumab responders or non-responders. RNA sequencing data of biopsies obtained before treatment (tumor and normal mucosa) were used for analysis. We used the “limma” package in R software to perform differential gene analysis and generate candidate genes. Additionally, we downloaded RNA sequencing data and clinical information for CRC patients from The Cancer Genome Atlas (TCGA) database. For the candidate genes generated from the GEO dataset, we further conducted Log-rank tests and multivariate Cox regression analyses in the TCGA database using R packages “survival” and “survminer”. All the figures were generated using R software.

### Plasmid construction

For overexpression of MAGEA3, the phage-3×HA-EGFP-puro plasmid encoding EGFP was gifted from Y&X Labs, and human MAGEA3 was then cloned into this vector. For the knockdown of MAGEA3, lentiviral vector pLKO.1 was used to construct a plasmid expressing short hairpin RNAs (shRNA) targeting MAGEA3 (pLKO.1-shMAGEA3-puro), and the negative control of shRNA (pLKO.1-shNC-puro, NC refers to negative control) was gifted by General Biotech, China.

### Lentivirus transfection

HEK293T cells were transfected with packaging vectors and the transfer plasmid using Polyethylenimine (PEI) (23966, Polysciences, USA). Then, the transfection medium was replaced with a fresh medium after transfection for 6 h. The HEK293T cells were further cultured for 48 h, and the medium containing lentivirus particles was collected for subsequent infection. To infect cells, the medium containing lentivirus and fresh medium were mixed equally and 8 μg/mL polybrene (TR-1003, Sigma-Aldrich, USA) was added to enhance infection efficiency. After 48 h of infection, cells were selected with 2 μg/mL (for HCT116) or 4 μg/mL (for HT29) puromycin for 3-4 days to obtain positive cells.

### Cell culture

The human colorectal cancer cell line HCT116 was purchased from the Cell Bank of the Chinese Academy of Sciences (Shanghai, China), and the human colorectal cancer cell line HT29 was obtained from Servicebio Technology, China (STCC10801P). Human colorectal cancer cell line Caco-2, human normal colon epithelial cell line FHC, and the HEK293T cells were gifted by Y&X Labs. HCT116 and HT29 cells were cultured in McCoy’s 5A Medium (L630KJ, BasalMedia Biotech, China), while Caco-2, FHC, and HEK293T cells were grown in Dulbecco’s Modified Eagle Medium (DMEM) (C11995500, Gibco, USA). Both media were supplemented with 10% fetal bovine serum (FBS) and 100 U/mL penicillin and 100 μg/mL streptomycin (S110JV, BasalMedia Biotech, China) unless otherwise stated. All cells were cultured in a humidified incubator at 37 ℃ with 5% CO_2_. DAPI staining was used to detect mycoplasma contamination of cells as previously described^[21]^. All the cell lines were confirmed to be mycoplasma-free before use in subsequent experiments.

### Western blot

Cells were lysed using mammalian cell lysis buffer (MCLB) with 1 × protease inhibitor cocktail (ThermoFisher) and 1 mM PMSF on ice for 30 min. Supernatants were collected after centrifugation at 12,000 rpm and at 4 ℃ for 20 min. Protein concentration was then measured using the Bradford method (5000205, BIO-RAD). Equal amounts of protein mixed with protein loading buffer were separated by 4%-20% SDS-PAGE gel and transferred to nitrocellulose filter membranes (HATF00010, Millipore, USA). After blocking with 5% skim milk in TBST, the membrane was incubated with different primary antibodies. Following TBST washes, the membrane was incubated with appropriate horseradish peroxidase (HRP)-conjugated secondary antibodies, and signals were then detected using an enhanced chemiluminescence (ECL) detection system (Sage Creation Science). The primary antibodies used are listed in Supplementary Table 1.

### RNA extraction and quantitative real-time PCR

For cultured cells, total RNA was extracted using the EZ-press RNA Purification Kit (B0004D, EZBioscience). For colon and cancer tissues, total RNA was extracted using the TRIzol method as previously described^[22]^. Then, 1 μg of RNA was reverse transcribed into cDNA using the HiScript III RT SuperMix for qRT-PCR Kit (R323-01, Vazyme). The cDNA was then used to measure mRNA levels of target genes using ChamQ SYBR qRT-PCR Master Mix (Q312-02, Vazyme). Finally, the results of quantitative real-time PCR (qPCR) were analyzed using the 2^ΔΔCT^ method.

### Seahorse assay

Mitochondrial oxygen consumption rate (OCR) was assessed using an Agilent Seahorse XF Cell Mito Stress Test Kit (Agilent Technologies, 103015-100), and glycolytic function was assessed with an Agilent Seahorse XF Glycolysis Stress Test Kit (Agilent Technologies, 103020-100). Briefly, HCT116 and HT29 cells (30,000/well) were seeded into wells of a Seahorse XF Cell Culture Microplate (Agilent Technologies, 102416-100). The medium was changed to 180 μL/well of Seahorse XF DMEM (Agilent Technologies, 103575-100) supplemented with 1mM pyruvate, 2 mM glutamine, and 25 mM glucose (for OCR) or Seahorse XF DMEM supplemented with 1 mM pyruvate and 2 mM glutamine (for ECAR) one hour before the assay. Then, the cell culture microplate was placed in a 37 ℃ non-CO_2_ incubator for 1 h. Different compounds were added into the appropriate ports of a hydrated sensor cartridge (OCR: 2 μM oligomycin, 0.5 μM FCCP, 0.5 μM rotenone and antimycin A; ECR: 10 mM glucose, 2 μM oligomycin, and 50 mM 2DG). Oxygen consumption and pH changes were analyzed using an Agilent Seahorse XFe/XF analyzer (XF96 pro, Agilent Technologies, USA) according to the manufacturer’s instructions.

### AOM/DSS-induced mice colon cancer model

AOM/DSS-induced carcinogenesis was performed as previously described^[23]^. In detail, eight-week-old male C57BL/6 mice were purchased from the Animal Laboratory, Shanghai Jiaotong University School of Medicine, Shanghai, China. For the azoxymethane (AOM, 0218397125, MPbio) and dextran sulfate sodium (DSS, 0216011080, MPbio)-induced carcinogenesis model, 10 mg/kg AOM was intraperitoneally injected on day 0. After 7 days, mice were treated with three courses of DSS treatment. In each course, mice were fed with 2.5% DSS solution or ddH_2_O as planned for 7 days and then recovered for two weeks. Two weeks after the last course, mice were euthanized. Then, colorectal tumor tissue, peri-tumor colon tissue in AOM/DSS-treated mice, and normal colon tissue in untreated mice were harvested for further experiments.

### Collection of clinical samples and IHC analysis

The clinical information and CRC tissue slices of 34 CRC patients who received bevacizumab after colonic or rectal surgery were collected in Renji Hospital Affiliated, Shanghai Jiao Tong University School of Medicine, Shanghai, China. Inclusion criteria included: (1) patients with colorectal cancer who received bevacizumab as part of their first-line therapy at stage IV; (2) availability of CRC tissue slices collected prior to bevacizumab treatment. Exclusion criteria included: the absence of disease progression assessment following bevacizumab treatment. Immunohistochemistry (IHC) of MAGEA3 and VEGF was performed as previously described^[24]^. IHC scores were calculated to analyze MAGEA3 and VEGF IHC staining^[25]^. Briefly, the intensity of staining was scored as follows (intensity score): 0 = no positive staining, 1 = weak positive staining, 2 = medium positive staining, 3 = strong positive staining. The proportion of positively stained cancer cells was scored as follows (proportion score): 0 = 0, 1 = 1%-25%, 2 = 26%-50%, 3 = 51%-75%, and 4 = 76%-100%. The IHC score was then calculated by multiplying the intensity score and the proportion score.

### Statistic methods

Graphed data are presented as the mean ± SD (Standard Deviation) unless otherwise stated. All experiments were repeated three times. Statistical analyses were performed using GraphPad Prism (version 8.0) software, with statistical significance assessed using a two-tailed unpaired Student’s *t*-test unless otherwise stated. (ns: no significance, ^*^P < 0.05, ^**^P < 0.01, ^***^P < 0.001, ^****^P < 0.0001)

## RESULTS

### Identification of potential biomarkers predicting bevacizumab efficacy in CRC

**Figure 1:**
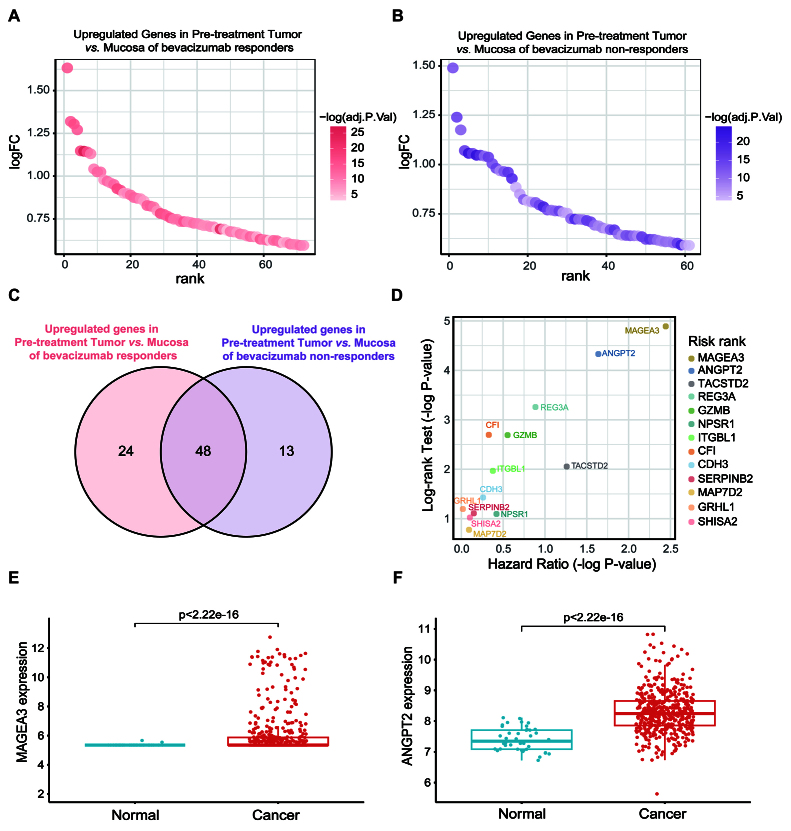
Identification of potential biomarkers predicting bevacizumab efficacy in CRC. (A) Scatter plot of upregulated differential expression genes (DEGs) in pre-treated colorectal cancer compared to mucosa in bevacizumab responders in dataset GSE60331. The DEGs were ranked in descending order by logFC along the X-axis. The color depth represents -log(adjusted *P*-value); (B) Scatter plot of upregulated differential expression genes (DEGs) in pre-treated colorectal cancer compared to mucosa in bevacizumab non-responders in dataset GSE60331. The DEGs were ranked in descending order by logFC along the X-axis. The color depth represents -log(adjusted *P*-value); (C) Venn diagram of DEGs in responders and non-responders; (D) Scatter plot of -log(*P*-value) from two survival analyses. The X-axis represents the -log(*P*-value) of the hazard ratio (HR) in the Cox proportional hazard model, and the Y-axis represents the -log(*P*-value) of the Log-rank test. The genes were marked with different colors and were ranked by risk to patient survival in descending order on the right side of the plot based on a combination of -log(*P*-value) values on the X-axis and Y-axis; (E and F) The expression of MAGEA3 (E) and ANGPT2 (F) in colorectal cancer and normal colon tissue in TCGA database. DEGs: differential expression genes; FC: fold change; HR: hazard ratio.

In order to identify potential biomarkers related to bevacizumab efficacy in CRC, we analyzed gene expression profiles of the GEO dataset GSE60331^[26]^. First, we screened genes that were significantly upregulated in pre-treatment tumor tissues compared to peri-tumor mucosa tissues in both bevacizumab responders and non-responders. Genes with a fold change (FC) > 1.5 and an adjusted *P*-value < 0.05 were selected. Our analysis identified 72 genes that were upregulated in pre-treatment tumors of responders [Figure 1A] and 61 genes that were upregulated in pre-treatment tumors of non-responders [Figure 1B]. A comparison of these gene sets revealed 13 genes that were exclusively upregulated in non-responders [Figure 1C, Supplementary Table 2]: Angiopoietin-2 (ANGPT2), CDH3, CFI, GRHL1, GZMB, ITGBL1, MAGEA3, MAP7D2, NPSR1, REG3A, SERPINB2, SHISA2, TACSTD2. To further assess the prognostic value of these genes, we analyzed their correlation with patient survival in CRC datasets from TCGA database using the Log-rank test and Cox proportional hazard model. Among these 13 genes, MAGEA3 and ANGPT2 showed significant correlations with patient survival in both statistical models (adjusted *P* < 0.05), with MAGEA3 displaying the most significant association [Figure 1D, Table 1]. In addition, in the TCGA database, both MAGEA3 and ANGPT2 were found to be significantly overexpressed in tumor tissues compared to normal tissues [Figure 1E and F]. In summary, we identified MAGEA3 and ANGPT2 as potential biomarkers for predicting bevacizumab efficacy in CRC, with MAGEA3 showing the most significant correlation with patient survival.

**Table 1:** Correlation and risk ranking of 13 hub genes with CRC patient survival in TCGA database

**ID**	**Log-rank_-log(*P* value)**	**HR_-log(*P* value)**	**Risk ranking**
*MAGEA3*	4.8900	2.4437	1
*ANGPT2*	4.3300	1.6383	2
*TACSTD2*	2.0540	1.2596	3
*REG3A*	3.2581	0.8861	4
*GZMB*	2.6900	0.5528	5
*NPSR1*	1.0952	0.4202	6
*ITGBL1*	1.9680	0.3768	7
*CFI*	2.6956	0.3279	8
*CDH3*	1.4285	0.2596	9
*SERPINB2*	1.1077	0.1487	10
*SHISA2*	1.0218	0.1024	11
*MAP7D2*	0.7749	0.0915	12
*GRHL1*	1.1951	0.0177	13

The -log(*P*-value) from the Log-rank test and Cox proportional hazard model were used to assess the prognostic risk of each gene. CRC: colorectal cancer; TCGA: The Cancer Genome Atlas; HR: hazard ratio.

### Exploring the expression of MAGEA3 in CRC

**Figure 2:**
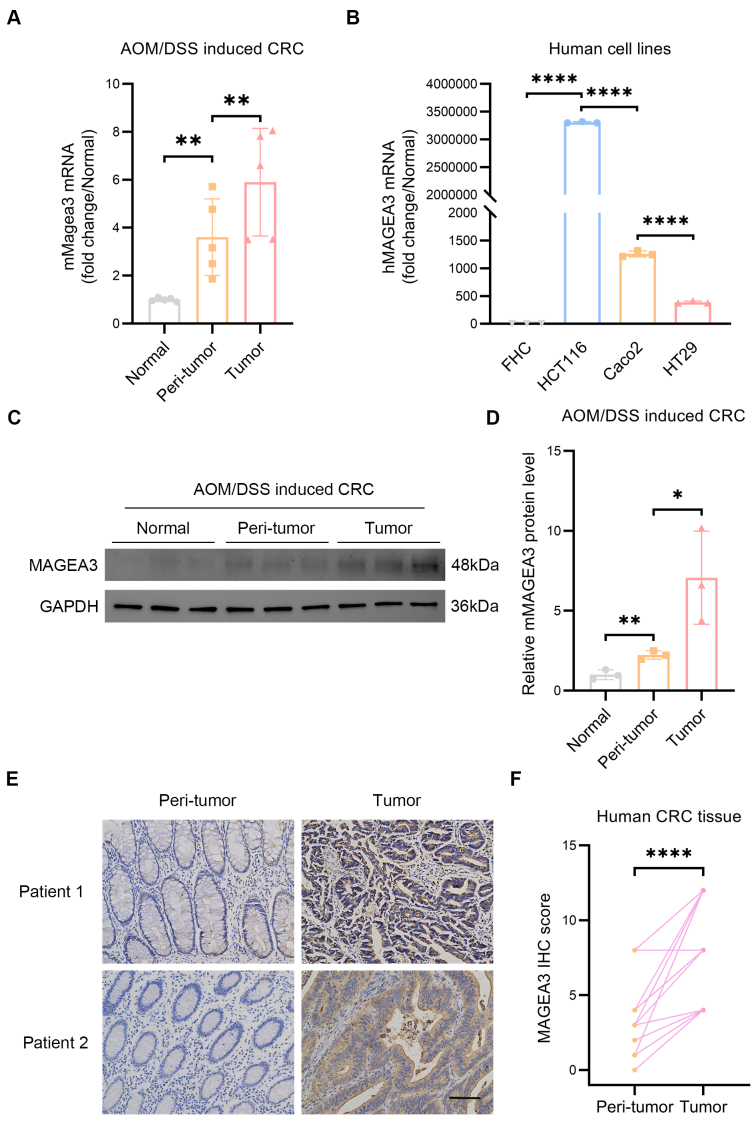
Exploring MAGEA3 expression in mice CRC and human CRC samples. (A) The mRNA level of MAGEA3 in tumor and peri-tumor colon tissues from AOM/DSS-treated mice compared to colon tissues in untreated mice; (B) The mRNA level of MAGEA3 in different human CRC cell lines HCT116, Caco-2, and HT29, compared to normal colon epithelial cell line FHC; (C and D) Images and quantification of Western blot of MAGEA3 in tumor and peri-tumor colon tissues from AOM/DSS-treated mice compared to colon tissues from untreated mice; (E and F) Representative images and quantification of immunohistochemistry staining of MAGEA3 in tumor and peri-tumor tissues of human CRC (scale bar: 100 μm). ns: no significance, ^*^P < 0.05, ^**^P < 0.01, ^****^P < 0.0001. CRC: colorectal cancer; AOM: azoxymethane; DSS: dextran sulfate sodium.

It has been reported that ANGPT2 plays a role in alternative angiogenic pathways, and both serum ANGPT2 levels and SNPs in tumors can predict bevacizumab resistance in some trials^[27,28]^. Building on this, our study primarily explores the role of MAGEA3 in resistance to antiangiogenic therapy. Since MAGEA3 is reported to have exclusively high expression in tumors, we assessed its expression in both AOM/DSS-induced mouse models of colorectal cancer and human colorectal cancer cell lines and tissues. Our results showed that the mRNA level of MAGEA3 in tumor and peri-tumor colon tissues from AOM/DSS-treated mice was significantly higher compared to normal colon tissues of untreated mice [Figure 2A]. In addition, the mRNA level of MAGEA3 was higher in all tested human CRC cell lines (HCT116, Caco-2 and HT29) than in the normal colon epithelial cell line (FHC) [Figure 2B]. Further, we used Western blot to detect the MAGEA3 protein level in AOM/DSS mouse models, which showed elevated protein level of MAGEA3 in the peri-tumor tissues and further elevation in the tumor tissues in AOM/DSS-treated mice compared to untreated mice [Figure 2C and D]. We also used IHC staining to detect the MAGEA3 protein level in human CRC samples. Results showed that the MAGEA3 protein level was higher in tumor tissue than in peri-tumor tissue in human CRC [Figure 2E and F]. Taken together, our results showed upregulated MAGEA3 expression in both AOM/DSS-induced mice models and human CRC samples.

### MAGEA3 modulates VEGF expression in CRC

**Figure 3:**
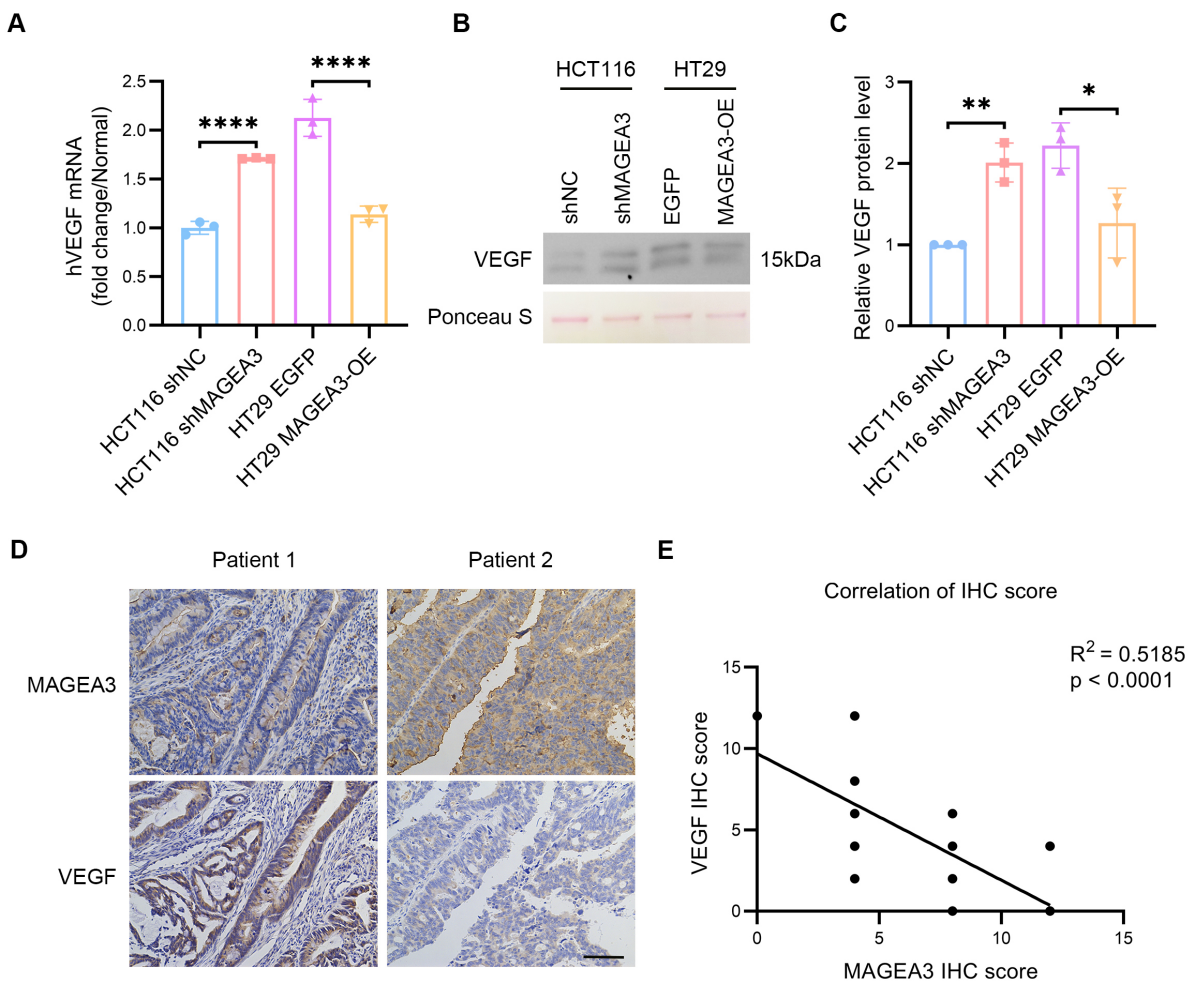
MAGEA3 inhibits VEGF expression in CRC. (A) The mRNA level of VEGF in the shNC and shMAGEA3 groups of HCT116 cells, and the EGFP and MAGEA3-OE groups of HT29 cells; (B and C) Representative images and quantification of Western blot of VEGF in medium of the shNC and shMAGEA3 groups of HCT116 cells, and the EGFP and MAGEA3-OE groups of HT29 cells cultured in serum-free medium for 24 h. Ponceau S staining was used as an internal loading control alongside Western blot detection of VEGF; (D) Representative immunohistochemistry (IHC) staining images of MAGEA3 and VEGF in human colorectal cancer (scale bar: 100 μm); (E) Correlation between the IHC scores of MAGEA3 and VEGF in human CRC. ^*^P < 0.05, ^**^P < 0.01, ^****^P < 0.0001. shNC: shRNA for negative control; MAGEA3-OE: MAGEA3 overexpression; IHC: immunohistochemistry.

Since the expression of MAGEA3 was the highest in HCT116 and the lowest in HT29 among human CRC cell lines mentioned above, we knocked down MAGEA3 in HCT116 and overexpressed MAGEA3 in HT29 cells to explore the mechanism related to MAGEA3. We utilized short hairpin RNA (shRNA) to knock down the expression of the MAGEA3 gene in HCT116 cells. The experimental group that received MAGEA3-specific shRNA was labeled as shMAGEA3 and the control group that received a non-targeting shRNA was labeled as shNC (shRNA for negative control). We also overexpressed the MAGEA3 gene in the experimental group (labeled as MAGEA3-OE, where OE refers to overexpression) and the enhanced green fluorescent protein (EGFP) gene in the control group (labeled as EGFP) in HT29 cells. Since VEGF is a major angiogenic factor produced by cancer cells and also the target of bevacizumab, we first investigated the impact of MAGEA3 on VEGF in cancer cells. The VEGF mRNA level increased following MAGEA3 knockdown in HCT116 cells, and decreased following MAGEA3 overexpression in HT29 cells [Figure 3A]. Further, Western blot was used to detect VEGF levels in the serum-free medium of the above cells, which showed that VEGF levels in the medium of HCT116 shMAGEA3 group were higher than those in the HCT116 shNC group, and VEGF levels in the HT29 MAGEA3-OE group were lower than those in HT29 EGFP group [Figure 3B and C]. In addition, immunohistochemical staining of human CRC tissue samples also showed a negative correlation between the IHC scores of MAGEA3 and VEGF [Figure 3D and E]. These results indicated that MAGEA3 can inhibit the expression and release of VEGF in cancer cells.

### mTOR pathway is involved in the regulation of VEGF by MAGEA3 in CRC

**Figure 4:**
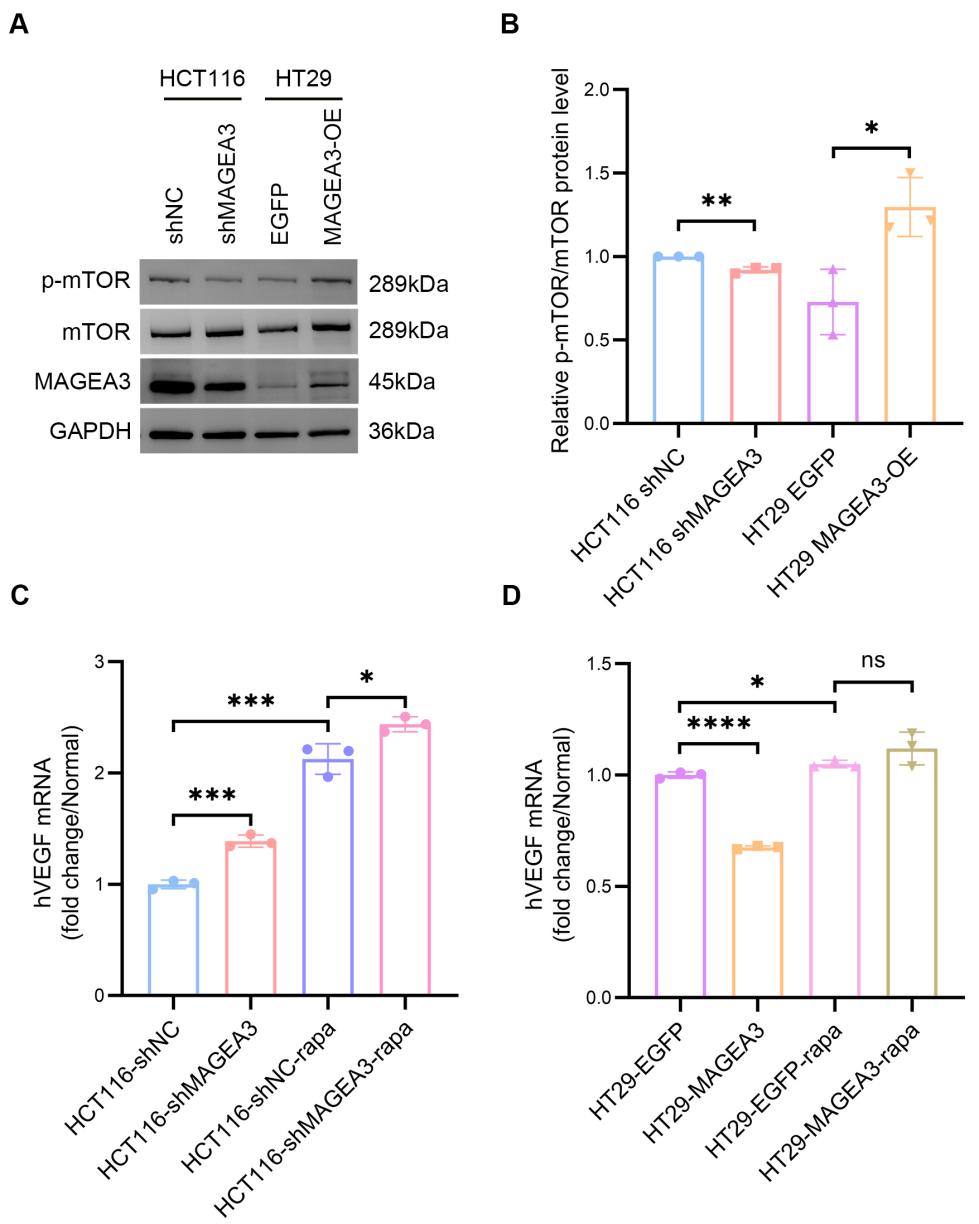
Rapamycin impacts the inhibitory effect of MAGEA3 on VEGF expression. (A and B) Western blot and quantification of phosphorylated mTOR protein level and total mTOR protein level after knocking down MAGEA3 in HCT116 cells and overexpression of MAGEA3 in HT29 cells; (C) mRNA levels of VEGF in shNC and shMAGEA3 HCT116 cell lines treated or untreated with rapamycin (rapa); (D) mRNA levels of VEGF in EGFP and MAGEA3 overexpression HT29 cell lines treated or untreated with rapamycin. ns: no significance, ^*^P < 0.05, ^**^P < 0.01, ^***^P < 0.001, ^****^P < 0.0001. shNC: shRNA for negative control; MAGEA3-OE: MAGEA3 overexpression; rapa: rapamycin.

We further investigated the potential pathway through which MAGEA3 may inhibit VEGF in CRC cells. Previous studies have suggested that MAGEA3 upregulates the mechanistic target of rapamycin (mTOR) pathway^[29]^. Our results also showed the phosphorylation of mTOR decreased in HCT116 cells after knocking down MAGEA3 and increased in HT29 cells after overexpression of MAGEA3 [Figure 4A and B]. We then tested whether MAGEA3 affects VEGF through the mTOR pathway by treating those cells with rapamycin (an inhibitor of the mTOR pathway). Results showed that in HCT116 cells, the VEGF expression in the shMAGEA3 group was higher than in the shNC group after rapamycin treatment, indicating that MAGEA3 depletion and rapamycin treatment regulated VEGF expression in the same direction [Figure 4C]. In HT29 cells, while VEGF expression in the MAGEA3-OE group was lower than in the EGFP group under untreated conditions, the difference disappeared after treatment with rapamycin, indicating that treatment with rapamycin reversed the inhibitory effect of MAGEA3 on VEGF expression [Figure 4D]. Taken together, these findings suggest that MAGEA3 inhibits VEGF expression by upregulating the mTOR pathway in CRC cells.

### MAGEA3 does not activate alternative angiogenic pathways in CRC

**Figure 5:**
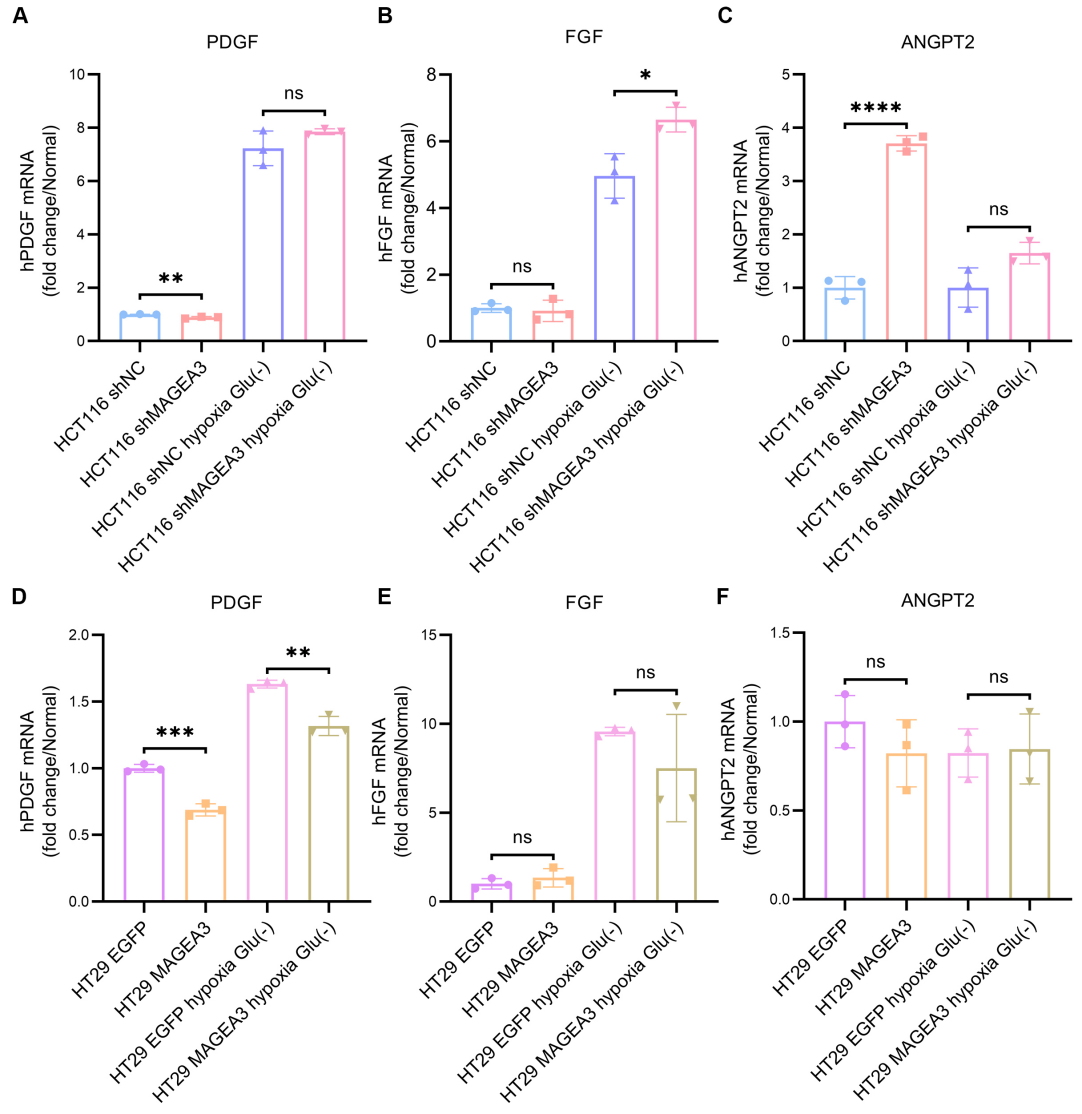
MAGEA3 does not activate PDGF, FGF and ANGPT2 in CRC cell lines. (A-C) mRNA levels of PDGF (A), FGF (B), and ANGPT2 (C) in shNC and shMAGEA3 HCT116 cell lines in normoxia and hypoxia & glucose-deprived (Glu(-)) conditions; (D-F) mRNA levels of PDGF (D), FGF (E), and ANGPT2 (F) in HT29 cell lines overexpressing EGFP and MAGEA3 in normoxia and hypoxia & glucose-deprived conditions. ns: no significance, ^*^P < 0.05, ^**^P < 0.01, ^***^P < 0.001, ^****^P < 0.0001. shNC: shRNA for negative control; MAGEA3-OE: MAGEA3 overexpression; Glu(-): glucose-deprived; PDGF: Platelet-derived growth factor; FGF: Fibroblast growth factor; ANGPT2: Angiopoietin-2.

Given that MAGEA3 inhibits VEGF expression and is highly expressed in bevacizumab-resistant patients, we further investigated whether MAGEA3 might activate other angiogenic pathways. As MAGEA3 is mainly expressed in CRC cells, which are key producers of various pro-angiogenic factors, we examined the correlation between MAGEA3 and major alternative angiogenic factors such as Platelet-derived growth factor (PDGF), Fibroblast growth factor (FGF), and ANGPT2^[9]^. Results showed that the mRNA levels of PDGF and FGF were not significantly affected after MAGEA3 knockdown in HCT116 under normoxia conditions, although the mRNA level of FGF was higher in the shMAGEA3 group in hypoxia & glucose-deprived conditions [Figure 5A and B]. The expression level of ANGPT2 increased after the knockdown of MAGEA3 in HCT116; however, the increase was suppressed under hypoxia & glucose-deprived conditions [Figure 5C]. Similarly, in HT29 cells, overexpression of MAGEA3 did not cause obvious changes in the expression of the aforementioned genes, although the mRNA level of PDGF was slightly lower in the MAGEA3-OE group [Figure 5D-F]. We further detected protein levels of PDGF, FGF, and ANGPT2 in serum-free medium of these cells after culturing in serum-free medium for 24 h. The levels of secreted PDGF and ANGPT2 were detectable in all HCT116 cells and HT29 cells, while secreted FGF was only detected in HCT116 cells treated with hypoxia & glucose-deprived conditions [Supplementary Figure 1A]. Additionally, knocking down and overexpression of MAGEA3 had a mild impact on secreted protein levels of both PDGF and ANGPT2 [Supplementary Figure 1B and C]. These results suggest that MAGEA3 does not activate the expression of alternative pro-angiogenic factors.

### MAGEA3 regulates the mitochondrial capacity of CRC cell lines

**Figure 6:**
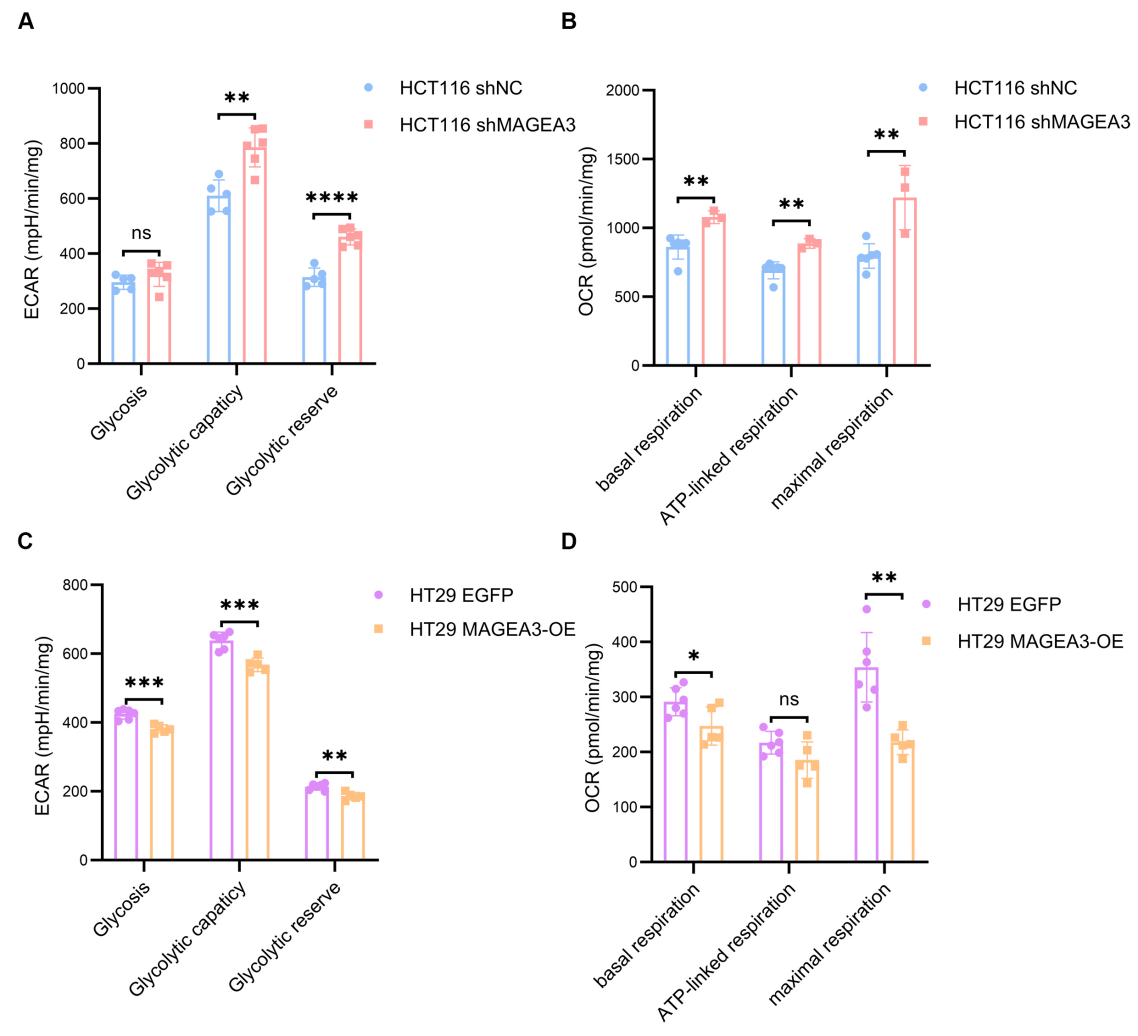
MAGEA3 regulates the mitochondrial capacity of CRC cell lines. (A and B) The extracellular acidification rate (A) and oxygen consumption rate (B) of shNC and shMAGEA3 HCT116 cell lines in Seahorse experiment; (C and D) The extracellular acidification rate (C) and oxygen consumption rate (D) of HT29 cell lines overexpressing EGFP and MAGEA3 in Seahorse experiment. ns: no significance, ^*^P < 0.05, ^**^P < 0.01, ^***^P < 0.001, ^****^P < 0.0001. shNC: shRNA for negative control; MAGEA3-OE: MAGEA3 overexpression.

Since our results suggested that MAGEA3 may have an inhibitory effect on angiogenesis, we explored whether MAGEA3 affected the metabolic capacity of CRC cells, enabling them to maintain a growth advantage even under conditions of relatively weak angiogenesis. Therefore, we used Seahorse experiments to detect the impact of MAGEA3 on the Extracellular Acidification Rate (ECAR) and Oxygen consumption rate (OCR) of HCT116 and HT29 cells. In HCT116 cells, although the ECAR assay showed no difference in glycolysis level between the shMAGEA3 and shNC groups, the shMAGEA3 group had higher glycolysis capacity and glycolysis reserve [Figure 6A]. OCR experiments showed that the knockdown of MAGEA3 increased the basal oxygen consumption, ATP-linked oxygen consumption, and maximum oxygen consumption of HCT116 cells [Figure 6B]. In HT29 cells, the MAGEA3-OE cell line had lower glycolysis level, glycolytic capacity, and glycolytic reserve than the EGFP control group [Figure 6C], as well as lower basal respiration and maximal respiration [Figure 6D]. These results indicate that MAGEA3 reduces the level of mitochondrial metabolism of CRC cells, which may provide a growth advantage for CRC cells under ischemic conditions.

### Prognostic value of MAGEA3 in CRC patients under bevacizumab treatment

**Figure 7:**
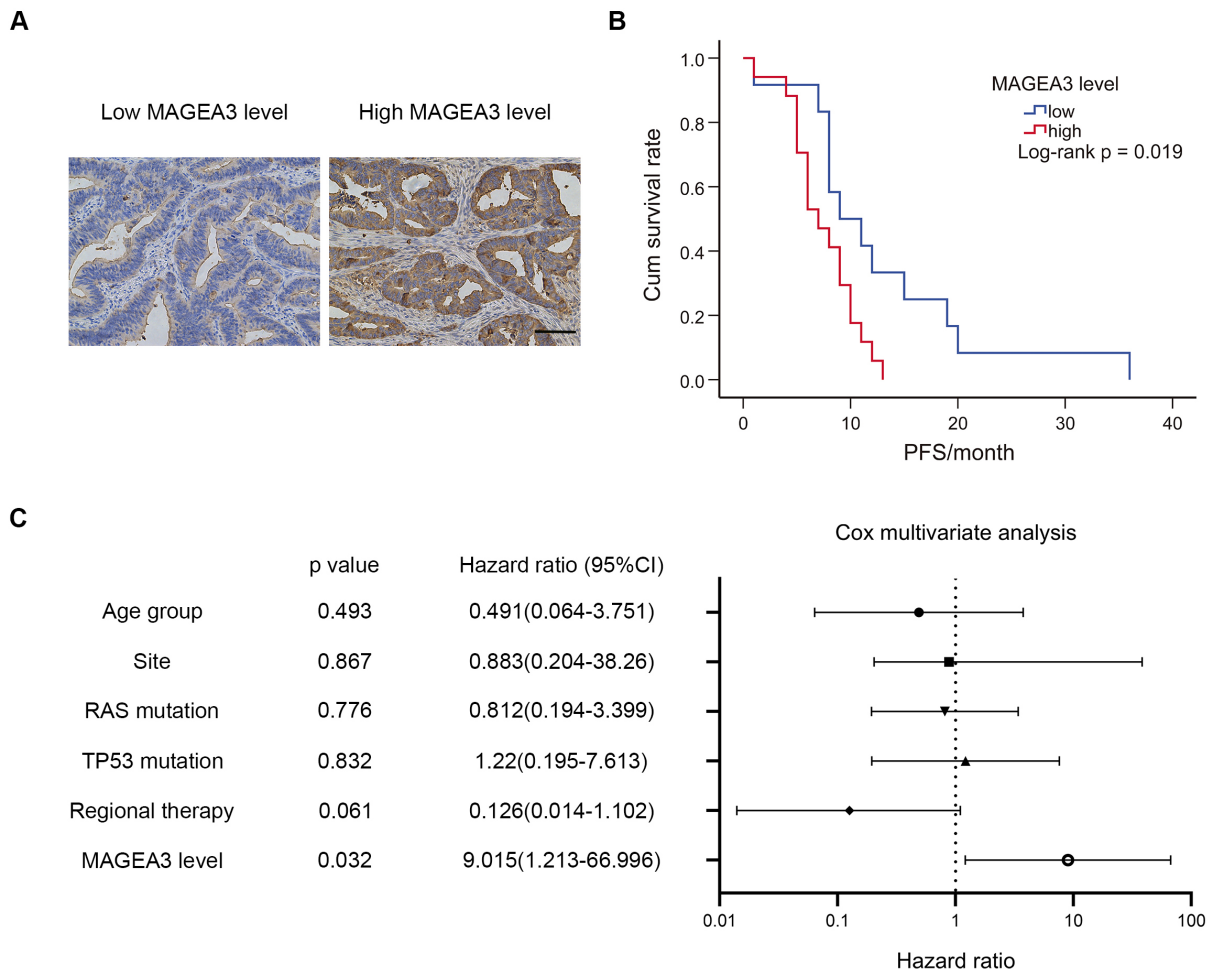
Validation of prognostic effect of MAGEA3 in CRC with bevacizumab treatment. (A) Representative immunohistochemistry staining (IHC) images of MAGEA3 in CRC slices with low MAGEA3 levels and high MAGEA3 levels (scale bar: 100 μm); (B) Kaplan-Meier survival analysis of progression-free survival (PFS) between CRC patients with high or low IHC scores of MAGEA3 (*P* = 0.019); (C) Cox multivariate analysis of PFS in the clinical cohort shown in Figure 7B. IHC: immunohistochemistry staining; PFS: progression-free survival; CI: confidence interval.

To validate the prognostic value of MAGEA3, we collected CRC tissue sections from 34 CRC patients prior to bevacizumab treatment. The common clinical characteristics of patients at the time of tissue collection were collected, including age, TNM stage, tumor location, RAS mutation status, TP53 mutation status, and use of regional therapy [Table 2]. Notably, 20 of 34 patients were at stage II or III when they underwent CRC surgery but began bevacizumab therapy after progressing to stage IV. MAGEA3 expression was detected in these slices using immunohistochemistry, and patients were divided into a low-expression group (IHC scores < 8) and a high-expression group (IHC scores ≥ 8) based on MAGEA3 IHC scores. Chi-square analysis showed no significant difference between patients with high and low MAGEA3 expression in the common clinical characteristics mentioned above. We then analyzed the correlation between MAGEA3 expression levels and progression-free survival (PFS) after bevacizumab treatment. Results showed that the median PFS of all patients was 8 months, with a 95% confidence interval (CI) ranging from 6.466 to 9.534 months. Patients with high MAGEA3 expression [7 months (95% CI: 4.908-9.420 months)] had shorter PFS than patients with low MAGEA3 expression [9 months (95% CI: 3.908-14.092 months)] [Figure 7A and B]. We further conducted a Cox multivariate analysis that included MAGEA3 levels and variations in Table 2 (except for TNM stage, as all the patients had already progressed to stage IV when they started bevacizumab therapy). Results showed that MAGEA3 was an independent risk factor for bevacizumab resistance [Hazard ratio 9.105 (95%CI: 1.213-66.996), *P* = 0.032] [Figure 7C]. Together, these results indicated that high MAGEA3 expression in CRC patients was associated with shorter progression-free survival (PFS) after bevacizumab treatment, suggesting its potential as a prognostic biomarker.

**Table 2:** Comparison of clinical characteristics between colorectal cancer patients with low and high MAGEA3 levels

**Variation**	**low MAGEA3 (*n* = 14)**	**high MAGEA3 (*n* = 20)**	***χ*^2^**	***P* value**
Age				
≤ 65	8	15	1.2	0.273
> 65	6	5		
TNM stage				
II	2	3	0.362	0.834
III	7	8		
IV	5	9		
Site				
Left colon	8	15	1.2	0.273
Right colon	6	5		
RAS mutation				
wild-type	5	9	0.201	0.654
mutated	7	9		
unknown	2	2		
TP53 mutation				
wild-type	4	3	0.949	0.622
mutated	5	8		
unknown	5	9		
Regional therapy				
No	8	11	0.015	0.901
Yes	6	9		

MAGEA3: Melanoma-associated antigen 3; TNM: tumor, node, metastasis; RAS: Rat sarcoma viral oncogene homolog; TP53: tumor protein 53 mutation.

## DISCUSSION

In this study, we identified MAGEA3 as a gene associated with primary resistance to bevacizumab in CRC patients using sequencing data, and further investigated its role in bevacizumab resistance and its impact on prognosis in CRC patients. We found that MAGEA3 in CRC cells inhibits the expression of VEGF through the mTOR pathway, but has no significant effect on other alternative angiogenic factors. Additionally, the high expression of MAGEA3 enables tumor cells to exhibit lower mitochondrial metabolism. Finally, we validated through clinical samples that CRC patients with high MAGEA3 levels had worse bevacizumab treatment efficacy.

Since MAGEA3 is aberrantly expressed in multiple tumors due to epigenetic regulation, several studies have suggested that it may play a key role in tumorigenesis^[14]^. In our AOM/DSS model, we found that MAGEA3 expression in peri-tumor colon tissue of AOM/DSS-treated mice was significantly higher than in colon tissue of untreated mice, further supporting the role of MAGEA3 in tumorigenesis. This characteristic makes MAGEA3 a potential biomarker for tumors in primary CRC sites. Previous studies have shown that high MAGEA3 levels are associated with worse prognosis in cutaneous squamous cell carcinoma, malignant peripheral nerve sheath tumor, and other cancers^[30,31]^. In our study, TCGA database analysis also revealed that CRC patients with high MAGEA3 levels exhibited worse prognosis.

Previous studies have shown that MAGEA3 plays diverse roles in tumor cell proliferation and apoptosis, and interacts with several signaling pathways. However, its potential involvement in resistance to antiangiogenic therapy remains unexplored. This study is the first to propose that MAGEA3 influences bevacizumab treatment efficacy through bioinformatic analysis and clinical validation. We then found that high expression of MAGEA3 reduces VEGF expression and secretion in cancer cells. Since bevacizumab inhibits the function of VEGF by competitively binding to it, our results suggest that MAGEA3 may contribute to bevacizumab resistance by reducing the number of targets available for bevacizumab inhibition. However, the predictive effect of VEGF on bevacizumab efficacy has been controversial in different clinical studies^[12,32]^, and VEGF was not exclusively upregulated in bevacizumab non-responders during bioinformatic screening. Together, this suggests that VEGF has high heterogeneity across different clinical trial populations, making it less suitable as a stable biomarker for clinical use. In contrast, MAGEA3 exhibited a more consistent correlation with bevacizumab resistance across different samples, suggesting that MAGEA3 may have a more stable prognostic effect than VEGF in CRC patients. It should be noted that MAGEA3 is only expressed in tumor cells, while VEGF can also be expressed by other stromal cells in cancer^[33]^. This may explain why MAGEA3 and VEGF, despite their negative regulatory relationship in tumor cells, do not fully correlate and have different prognostic effects in CRC populations.

For tumors with high MAGEA3 expression, while reduced VEGF expression may partially explain bevacizumab resistance, it is necessary to explore how CRC cells can continue growing under conditions of relatively low VEGF. We proposed two hypotheses based on the known mechanisms of antiangiogenic drug resistance and the functions of MAGEA3: (1) Although MAGEA3 inhibits VEGF expression, it may activate other alternative angiogenic factors to maintain angiogenesis and provide adequate nutrition; (2) MAGEA3 may alter the metabolic state of tumor cells, enabling them to continue growing under ischemic conditions. In our study, results showed that MAGEA3 did not upregulate the expression of PDGF, FGF, and ANGPT2 but could lower glycolysis and respiration levels in cancer cells. These results suggest that MAGEA3 might enhance CRC cells’ adaptability by reducing their energy metabolism demands. Previous studies have also shown that MAGEA3 can regulate the ubiquitination degradation of AMPK, as well as activate the mTOR pathway and inhibit the autophagy pathway^[29]^, which can also impact the metabolic status of tumor cells. This result, together with our findings, indicates that MAGEA3 may provide a growth advantage for tumor cells under ischemic conditions by lowering their nutritional demands, which can eventually contribute to bevacizumab resistance.

Because of the tumor-specific expression patterns and pro-tumorigenic functions of MAGEA3, immunotherapy and targeted therapy have been explored in previous studies. The initial identification of MAGEA3 as a tumor neoantigen in melanoma and non-small cell lung cancer (NSCLC) has prompted many MAGEA3-based cancer vaccines and adoptive T cell therapy studies^[34,35]^. However, these therapies failed to increase survival in phase III clinical trials, which highlights a critical need for a more detailed understanding of the underlying mechanisms^[36,37]^. Compared to immunotherapy, research on drugs directly targeting MAGEA3 is still in its early stages. Ke Li *et al.* developed proteolysis-targeting chimera (PROTAC) molecules that can induce MAGEA3 degradation, which inhibits the proliferation of some MAGEA3-positive cell lines^[38]^. As the mTOR pathway is activated in colorectal cells with high MAGEA3 levels, rapamycin may have the potential therapeutic efficacy as an indirect targeting approach against MAGEA3-high CRC. The combination of a MAGEA3 inhibitor and rapamycin may have a synergetic effect, which requires further investigation.

There are some limitations that should be acknowledged in our study. First, while we focused on establishing the biomarker potential of MAGEA3, mechanistic studies using functional mouse models would provide additional insights into how MAGEA3 influences bevacizumab efficacy in the tumor microenvironment, and may open a new avenue for targeting MAGEA3 in cancer treatment. Second, given that class I MAGE proteins often show coordinated expression patterns^[13,14]^, investigating potential interactions between MAGEA3 and other family members could be informative. Finally, while our clinical cohort demonstrated an association between MAGEA3 and treatment outcome, larger prospective studies would help further characterize its prognostic value.

In conclusion, our study explored the relationship between MAGEA3 and bevacizumab resistance in primary CRC and proposed potential mechanisms underlying this association. Our findings suggest that MAGEA3 could serve as a potential biomarker for bevacizumab resistance and patient prognosis in CRC.

## DECLARATIONS

### Availability of data and materials

The rata data supporting the findings of this study are available within this Article and its Supplementary Information. Further data are available from the corresponding authors upon reasonable request.

### Copyright

© The Author(s) 2025.

## References

[1] Siegel RL, Miller KD, Fuchs HE, Jemal A (2022). Cancer statistics, 2022. CA Cancer J Clin.

[2] Cao Y, Langer R, Ferrara N (2023). Targeting angiogenesis in oncology, ophthalmology and beyond. Nat Rev Drug Discov.

[3] Hanahan D, Weinberg RA (2000). The hallmarks of cancer. Cell.

[4] Ellis LM, Hicklin DJ (2008). Pathways mediating resistance to vascular endothelial growth factor-targeted therapy. Clin Cancer Res.

[5] Ince WL, Jubb AM, Holden SN (2005). Association of k-ras, b-raf, and p53 status with the treatment effect of bevacizumab. J Natl Cancer Inst.

[6] Cervantes A, Adam R, Roselló S, ESMO Guidelines Committee (2023). Electronic address: clinicalguidelines@esmo.org. Metastatic colorectal cancer: ESMO Clinical Practice Guideline for diagnosis, treatment and follow-up. Ann Oncol.

[7] Chen G (2017). Interpretation of the updates of NCCN 2017 version 1.0 guideline for colorectal cancer. Chin J Gastrointest Surg.

[8] Haibe Y, Kreidieh M, El Hajj H (2020). Resistance mechanisms to anti-angiogenic therapies in cancer. Front Oncol.

[9] Yang Z, Zhang X, Bai X, Xi X, Liu W, Zhong W (2024). Anti-angiogenesis in colorectal cancer therapy. Cancer Sci.

[10] Zheng Y, Zhou R, Cai J (2023). Matrix stiffness triggers lipid metabolic cross-talk between tumor and stromal cells to mediate bevacizumab resistance in colorectal cancer liver metastases. Cancer Res.

[11] Lambrechts D, Lenz HJ, de Haas S, Carmeliet P, Scherer SJ (2013). Markers of response for the antiangiogenic agent bevacizumab. J Clin Oncol.

[12] Jubb AM, Hurwitz HI, Bai W (2006). Impact of vascular endothelial growth factor-A expression, thrombospondin-2 expression, and microvessel density on the treatment effect of bevacizumab in metastatic colorectal cancer. J Clin Oncol.

[13] Simpson AJ, Caballero OL, Jungbluth A, Chen YT, Old LJ (2005). Cancer/testis antigens, gametogenesis and cancer. Nat Rev Cancer.

[14] Doyle JM, Gao J, Wang J, Yang M, Potts PR (2010). MAGE-RING protein complexes comprise a family of E3 ubiquitin ligases. Mol Cell.

[15] Kim KH, Choi JS, Kim IJ, Ku JL, Park JG (2006). Promoter hypomethylation and reactivation of MAGE-A1 and MAGE-A3 genes in colorectal cancer cell lines and cancer tissues. World J Gastroenterol.

[16] Wu F, Liu F, Dong L (2018). miR-1273g silences MAGEA3/6 to inhibit human colorectal cancer cell growth via activation of AMPK signaling. Cancer Lett.

[17] Xie C, Subhash VV, Datta A (2016). Melanoma associated antigen (MAGE)-A3 promotes cell proliferation and chemotherapeutic drug resistance in gastric cancer. Cell Oncol (Dordr).

[18] Gao X, Li Q, Chen G, He H, Ma Y (2020). MAGEA3 promotes proliferation and suppresses apoptosis in cervical cancer cells by inhibiting the KAP1/p53 signaling pathway. Am J Transl Res.

[19] Das B, Senapati S (2019). Functional and mechanistic studies reveal MAGEA3 as a pro-survival factor in pancreatic cancer cells. J Exp Clin Cancer Res.

[20] Liu XL, Zhao D, Sun DP (2012). Adenovirus-mediated delivery of CALR and MAGE-A3 inhibits invasion and angiogenesis of glioblastoma cell line U87. J Exp Clin Cancer Res.

[21] Uphoff CC, Gignac SM, Drexler HG (1992). Mycoplasma contamination in human leukemia cell lines. I. Comparison of various detection methods. J Immunol Methods.

[22] Jiahui W, Xiang Y, Youhuan Z (2025). The mitochondrial DNAJC co-chaperone TCAIM reduces α-ketoglutarate dehydrogenase protein levels to regulate metabolism. Mol Cell.

[23] Schepelmann M, Kupper N, Gushchina V (2022). AOM/DSS induced colitis-associated colorectal cancer in 14-month-old female Balb/C and C57/Bl6 mice-a pilot study. Int J Mol Sci.

[24] Li W, Huang M, Wu Z (2024). mRNA-lipid nanoparticle-mediated restoration of PTPN14 exhibits antitumor effects by overcoming anoikis resistance in triple-negative breast cancer. Adv Sci (Weinh).

[25] Li W, Lan J, Zhou C (2024). Chromosomal instability is associated with prognosis and efficacy of bevacizumab after resection of colorectal cancer liver metastasis. Ann Med.

[26] Verstraete M, Debucquoy A, Dekervel J (2015). Combining bevacizumab and chemoradiation in rectal cancer. Translational results of the AXEBeam trial. Br J Cancer.

[27] Goede V, Coutelle O, Neuneier J (2010). Identification of serum angiopoietin-2 as a biomarker for clinical outcome of colorectal cancer patients treated with bevacizumab-containing therapy. Br J Cancer.

[28] Stremitzer S, Zhang W, Yang D (2015). Genetic variations in angiopoietin and pericyte pathways and clinical outcome in patients with resected colorectal liver metastases. Cancer.

[29] Pineda CT, Ramanathan S, Fon Tacer K (2015). Degradation of AMPK by a cancer-specific ubiquitin ligase. Cell.

[30] Chen A, Santana AL, Doudican N (2020). MAGE-A3 is a prognostic biomarker for poor clinical outcome in cutaneous squamous cell carcinoma with perineural invasion via modulation of cell proliferation. PLoS One.

[31] Nobeyama Y, Nakagawa H (2016). MAGEA3 methylation status is associated with prognosis of malignant peripheral nerve sheath tumor and with neurofibroma type in neurofibromatosis type 1. J Dermatol Sci.

[32] Garcia J, Hurwitz HI, Sandler AB (2020). Bevacizumab (Avastin^®^) in cancer treatment: a review of 15 years of clinical experience and future outlook. Cancer Treat Rev.

[33] Ghalehbandi S, Yuzugulen J, Pranjol MZI, Pourgholami MH (2023). The role of VEGF in cancer-induced angiogenesis and research progress of drugs targeting VEGF. Eur J Pharmacol.

[34] Kruit WH, Suciu S, Dreno B (2013). Selection of immunostimulant AS15 for active immunization with MAGE-A3 protein: results of a randomized phase II study of the European Organisation for Research and Treatment of Cancer Melanoma Group in Metastatic Melanoma. J Clin Oncol.

[35] Lu YC, Parker LL, Lu T (2017). Treatment of patients with metastatic cancer using a major histocompatibility complex class II-restricted T-cell receptor targeting the cancer germline antigen MAGE-A3. J Clin Oncol.

[36] Vansteenkiste JF, Cho BC, Vanakesa T (2016). Efficacy of the MAGE-A3 cancer immunotherapeutic as adjuvant therapy in patients with resected MAGE-A3-positive non-small-cell lung cancer (MAGRIT): a randomised, double-blind, placebo-controlled, phase 3 trial. Lancet Oncol.

[37] Dreno B, Thompson JF, Smithers BM (2018). MAGE-A3 immunotherapeutic as adjuvant therapy for patients with resected, MAGE-A3-positive, stage III melanoma (DERMA): a double-blind, randomised, placebo-controlled, phase 3 trial. Lancet Oncol.

[38] Li K, Krone MW, Butrin A, Bond MJ, Linhares BM, Crews CM (2024). Development of ligands and degraders targeting MAGE-A3. J Am Chem Soc.

