# Supplementary material for: Unveiling MAGEA3: a novel predictive biomarker for bevacizumab resistance in colorectal cancer
Source: Cancer Drug Resist. 2025 Apr 28;8:22. doi: 10.20517/cdr.2025.35 (PMC12059477; doi:10.20517/cdr.2025.35)
Supplement: Supplementary file 1 [file cdr-8-22-SupplementaryMaterials.pdf]

Supplementary Materials

Unveiling MAGEA3: a novel predictive biomarker for bevacizumab resistance in colorectal cancer

Juncheng Su[1], Jiahui Wang[2], Weilin Chen[1], Yingjie Xu[2], Wen Yang[2], Weiwei Liu[2], Zheng Wang[1], Masha Huang[2]

[1]Department of Gastrointestinal Surgery, Renji Hospital Affiliated, Shanghai Jiao Tong University School of Medicine, Shanghai 200127, China.
[2]Department of Biochemistry and Molecular Cell Biology, Shanghai Key Laboratory for Tumor Microenvironment and Inflammation, Shanghai Jiao Tong University School of Medicine, Shanghai 200025, China.

Correspondence to: Prof./Dr. Masha Huang and Prof./Dr. Weiwei Liu, Department of Biochemistry and Molecular Cell Biology, Shanghai Key Laboratory for Tumor Microenvironment and Inflammation, Shanghai Jiao Tong University School of Medicine, 227 South Chongqing Road, Shanghai 200025, China. E-mail: martha0126@shsmu.edu.cn, liuweiwei08@163.com; Prof./Dr. Zheng Wang, Department of Gastrointestinal Surgery, Renji Hospital Affiliated, Shanghai Jiao Tong University School of Medicine, 160 Pujian Road, Shanghai 200127, China. E-mail: wangzh1972@126.com

Supplementary Table 1. Information of primary antibodies used for Western blots

Antibody	Category number	Company
anti-MAGEA3	60054-1-Ig	Proteintech
anti-VEGF	65373	Cell Signaling Technology
anti-GAPDH	60004-1-Ig	Proteintech
anti-p-mTOR	2974	Cell Signaling Technology
anti-mTOR	2983	Cell Signaling Technology
anti-PDGFA	26940-1-AP	Proteintech
anti-FGF1	17400-1-AP	Proteintech
anti-ANGPT2	24613-1-AP	Proteintech

Supplementary Table 2. Information of 13 hub genes exclusively upregulated in pre-treatment tumor tissue of bevacizumab non-responders in Figure 1

Differential expression analysis of pre-treatment Tumor *vs*. Mucosa in bevacizumab non-responders		
	logFC	adj.*P*.Val
ANGPT2	0.621	1.73E-07
CDH3	0.592	5.26E-10
CFI	0.621	1.23E-06
GRHL1	0.669	6.57E-07
GZMB	0.603	3.56E-05
ITGBL1	0.608	1.01E-04
MAGEA3	0.814	1.86E-02
MAP7D2	0.639	6.60E-07
NPSR1	0.590	7.57E-03
REG3A	0.885	5.07E-03
SERPINB2	0.592	6.58E-03
SHISA2	0.769	3.26E-05
TACSTD2	0.981	1.25E-07

FC: fold change; adj.*P*.Val: adjusted *P* value.

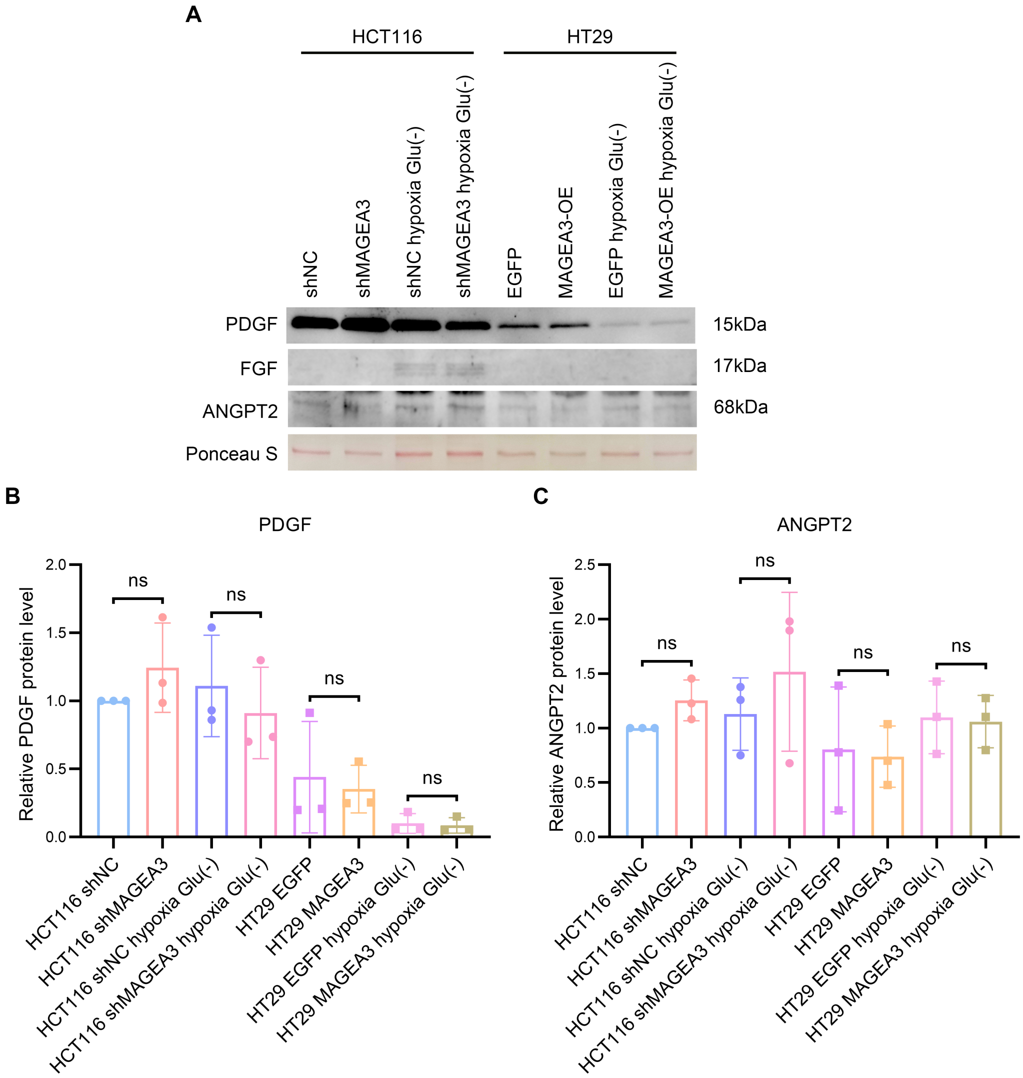

Supplementary Figure 1. MAGEA3 does not increase the secreted protein levels of PDGF, FGF and ANGPT2 in CRC cell lines. (A) Western blot images of PDGF, FGF and ANGPT2 in different serum-free media from the shNC, shMAGEA3 group of HCT116 cells and the EGFP, MAGEA3-OE group of HT29 cells after treatment for 24 hours in normoxia and hypoxia & glucose-deprived (Glu(-)) conditions. Ponceau S staining was used as an internal loading control alongside Western blot; (B) Quantification of Western blot of PDGF in Figure S1A; (C) Quantification of Western blot of ANGPT2 in Figure S1A. ns: no significance. shNC: shRNA for negative control; MAGEA3-OE: MAGEA3 overexpression; Glu(-): glucose-deprived. PDGF: Platelet-derived growth factor; FGF: Fibroblast growth factor; ANGPT2: Angiopoietin-2.
